# Identification of diagnostic markers in colorectal cancer via integrative epigenomics and genomics data

**DOI:** 10.3892/or.2015.3993

**Published:** 2015-05-19

**Authors:** TEOW KOK-SIN, NORFILZA MOHD MOKHTAR, NUR ZARINA ALI HASSAN, ISMAIL SAGAP, ISA MOHAMED ROSE, ROSLAN HARUN, RAHMAN JAMAL

**Affiliations:** 1 UKM Medical Molecular Biology Institute, Faculty of Medicine, Universiti Kebangsaan Malaysia, Kuala Lumpur, Malaysia; 2 Department of Physiology, Faculty of Medicine, Universiti Kebangsaan Malaysia, Kuala Lumpur, Malaysia; 3 Department of Surgery, Faculty of Medicine, Universiti Kebangsaan Malaysia, Kuala Lumpur, Malaysia; 4 Department of Pathology, Faculty of Medicine, Universiti Kebangsaan Malaysia, Kuala Lumpur, Malaysia; 5 Department of Medicine, Faculty of Medicine, Universiti Kebangsaan Malaysia, Kuala Lumpur, Malaysia

**Keywords:** colorectal cancer, epigenetics, DNA methylation, CpG loci, gene expression

## Abstract

Apart from genetic mutations, epigenetic alteration is a common phenomenon that contributes to neoplastic transformation in colorectal cancer. Transcriptional silencing of tumor-suppressor genes without changes in the DNA sequence is explained by the existence of promoter hypermethylation. To test this hypothesis, we integrated the epigenome and transcriptome data from a similar set of colorectal tissue samples. Methylation profiling was performed using the Illumina InfiniumHumanMethylation27 BeadChip on 55 paired cancer and adjacent normal epithelial cells. Fifteen of the 55 paired tissues were used for gene expression profiling using the Affymetrix GeneChip Human Gene 1.0 ST array. Validation was carried out on 150 colorectal tissues using the methylation-specific multiplex ligation-dependent probe amplification (MS-MLPA) technique. PCA and supervised hierarchical clustering in the two microarray datasets showed good separation between cancer and normal samples. Significant genes from the two analyses were obtained based on a ≥2-fold change and a false discovery rate (FDR) P-value of <0.05. We identified 1,081 differentially hypermethylated CpG sites and 36 hypomethylated CpG sites. We also found 709 upregulated and 699 downregulated genes from the gene expression profiling. A comparison of the two datasets revealed 32 overlapping genes with 27 being hypermethylated with downregulated expression and 4 hypermethylated with upregulated expression. One gene was found to be hypomethylated and downregulated. The most enriched molecular pathway identified was cell adhesion molecules that involved 4 overlapped genes, *JAM2*, *NCAM1*, *ITGA8* and *CNTN1*. In the present study, we successfully identified a group of genes that showed methylation and gene expression changes in well-defined colorectal cancer tissues with high purity. The integrated analysis gives additional insight regarding the regulation of colorectal cancer-associated genes and their underlying mechanisms that contribute to colorectal carcinogenesis.

## Introduction

In recent decades, the incidence of colorectal cancer (CRC) has increased by 2- to 4-fold in many Eastern Asia countries such as China, Japan, South Korea and Singapore (1,2). The high risk of CRC among the Asian population, including Malaysia, is associated with a low fiber diet and high tobacco consumption (3). One of the screening methods to detect early stage of CRC is by measuring the level of carcinoembryonic antigen (CEA) in the serum, however, the sensitivity of the marker was reported to be <80% (4–6). Therefore, the identification of new biological markers for CRC is crucial.

Epigenetic markers such as methylation markers in CRC were first reported 10 years ago in DNA from stool and blood samples (7). DNA methylation is an epigenetic mechanisms that involves the enzymatic process of adding the methyl group to the 5-carbon position of the cytosine to form 5-meth-ylcytosine (8–10). This modification mostly occurs in the CG enriched site known as CpG islands (CGI), which are present in 70% of the annotated gene promoter regions (11). Two typical DNA methylation patterns, global hypomethylation and CGI hypermethylation, have emerged as potential signatures in the cancer genome (12). Hypermethylation is generally found in the promoter CGI region, whereas global hypomethylation frequently occurs in CpG dinucleotides that are located in the repetitive sequences of DNA (satellite repeats or retrotransposon) (13).

CGI hypermethylation in the promoter region is thought to be linked with the transcriptional inactivation of tumor-suppressor genes (14,15). This is mediated through the methyl-CpG binding proteins (MBDs) resulting in a compacted chromatin conformation (15,16). The compacted chromatin hinders the accessibility of the transcriptional machinery from binding to the promoter region, thereby leading to the repression of gene expression (17). The inverse relationship between DNA methylation and transcript level was reported in a study involving chromosomes 6, 20 and 22 in 43 healthy human tissue and primary cells (18). The study revealed that one-third of the differentially methylated genes (representing 17% of the 873 analyzed genes) were found to be inversely associated with their transcript levels (18).

Apart from the conventional polymerase chain reaction (PCR) method, the microarray chip-based study is a widely used approach in exploring methylation markers in CRC (14,19,20). In a study using the Illumina GoldenGate^®^ methylation array on 28 normal mucosa and 91 CRC samples, 202 CpG sites with 90 hypermethylated and 42 hypomethylated loci that involved 132 genes were identified (21). Using the level of CpG island methylator phenotype (CIMP), CRC was divided into three different subgroups (21). A more recent study identified 169 hypermethylated loci and validated 11 of these loci that could be distinguished between CRC and non-neoplastic colonic mucosa (22). Among the genes were dedicator of cytokinesis 8 (*DOCK8*), visual system homeobox 2 (*VSX2*), microRNA 34b (*miR-34b*), glucagon-like peptide 1 receptor (*GLP1R1*), B-cell translocation gene 4 (*BTG4*), BEN domain containing 4 (*BEND4*), neuronal pentraxin I (*NPTX1*), ALX homeobox 3 (*ALX3*), zinc finger protein 583 (*ZNF583*), homer homolog 2 (*HOMER2*) and gap junction protein, gamma 1 (*GJC1*) (22).

Hypermethylated markers identified previously in CRC using a single array profile cannot reflect completely the complexity of the disease. To address this issue, epigenomic and genomic data from microarray analyses have been used to obtain a more comprehensive insight of the molecular mechanisms involved in CRC (17,23–25). The Cancer Genome Atlas study revealed a comprehensive molecular image of CRC by integrating data between promoter methylation, DNA copy number, exome sequencing, microRNA expression and messenger RNA expression in 224 CRC and normal samples (26). Apart from identifying gene mutations involved in the well-established signaling pathways, the authors of that study also documented the new critical role of *MYC* in directing the transcriptional activation and repression in CRC. Additional findings included repetitive mutations in APC membrane recruitment protein 1 (*FAM123B*) and AT rich interactive domain 1A *(ARID1A*) as well as a novel mutation in SRY (sex determining region Y)-box 9 (*SOX9*) (26).

Studies using a similar integrative approach of analyses on Asian patients are lacking. Therefore, the aim of the present study was to investigate the biological complexity of CRC by integrating DNA methylation and gene expression profiling signatures using paired samples from local patients. The integrated signatures may later serve as potential diagnostic markers for the prognostication of CRC. We also hypothesized that CpG hypermethylation in the promoter region of CRC-associated genes led to a low expression of the respective genes.

## Materials and methods

### Clinical samples

A total of 55 paired colorectal carcinoma and their corresponding adjacent normal epithelial cells (10 cm away from the tumor) were collected at surgery from patients at the Universiti Kebangsaan Malaysia Medical Centre, Kuala Lumpur, Malaysia. Patients provided written informed consent to participate in the present study. This study was approved by the UKM Research Ethics Committee (Reference no: UKM 1.5.3.5/244/UMBI-004-2012).

### Sample preparation

The clinical tissues were snap-frozen in liquid nitrogen prior to sectioning. Tissue samples were sectioned into 5–7 *μ*m thickness using a cryostat (Microtome Cryostat HM550; Microm International GmbH, Walldorf, Germany). The sections were stained with hematoxylin and eosin (H&E) and were examined by the histopathologist. Tissue samples were considered representative when >80% malignant cells were present. Normal cells also contained >80% normal epithelial cells and were free from malignant or inflammatory cells. Patients with chemotherapy or radiotherapy prior to surgery were excluded from the study.

### DNA methylation profiling assay

Genomic DNA was isolated using the Qiagen DNeasy Tissue kit (Qiagen, Hilden, Germany) according to the manufacturer’s instructions. The quantity and purity of DNA were quantified using the NanoDrop ND-1000 spectrophotometer (Thermo Fisher Scientific, Leicester, UK). Gel electrophoresis was used to check the integrity of the DNA. Only samples with good purity were included in the study. Methylation profiling was performed in 110 samples using the HumanMethylation27 Beadchip to analyze 27,578 CpG sites covering 14,495 genes. Bisulphite conversion was carried out in the methylation assay using the EZ DNA Methylation-Gold kit (Zymo Research, Irvine, CA, USA). The microarray study was carried out according to the Infinium II Methylation Assay manual protocol. All the chips were scanned on a single BeadArray reader to avoid bias.

### Gene expression profiling assay

Total RNA from 15 paired representative tissues were extracted using the Qiagen QIAampMini Plus kit (Qiagen) according to the manufacturer’s instructions. The quantity and purity of RNA were quantified using the NanoDrop ND-1000 spectrophotometer (Thermo Fisher Scientific). The integrity of RNA was measured using the Agilent Bioanalyzer 2100 (Agilent Technologies, Santa Clara, CA, USA). Only samples with an OD 260/280 of 1.8–2.1 and RNA integrity number (RIN) ≥6.0 were included in the gene expression study. The GeneChip Human Gene 1.0 ST array (Affymetrix, Santa Clara, CA, USA) that has 764,885 distinct probes covering 28,869 well-annotated genes was used. The assay was carried out using the Affymetrix expression protocol. The microarray chips were scanned using the GeneChip Scanner 3000 7G (Affymetrix). The Affymetrix^®^ Genotyping Console™ (Affymetrix) was used to extract the expression data and Partek Genomic Suite 6.6 (version 6.12.0713) (Partek Inc., St. Louis, MO, USA) was utilized for subsequent analysis.

### Statistical analysis of DNA methylation profiling

The control panel from Illumina BeadStudio software 2011 (version 1.9.0) (Illumina, San Diego, CA, USA) was used to determine the quality of our methylation microarray assay. β-value was used to determine the methylation status of each sample. This value was derived from each locus from the microarray and range from the lowest methylation value (β=0) to the highest methylation value (β=1). β-value was generated as the intensity of the methylated probe/total of the intensity of the methylated probe and the intensity of the unmethylated probe.

The generated β-value was exported to the Partek Genomic Suite 6.6 (version 6.12.0713) (Partek) for subsequent analysis. PCA mapping was used to determine the quality of the samples. Three-way analysis of variance (ANOVA) with ≥2-fold change and P<0.05 with FDR were used to compare the differential methylated CpG loci between the cancer and normal groups. To remove the batch effect, the scanned date of the chips was controlled. A Gene Ontology enrichment analysis was carried out to investigate whether the genes found to be differentially methylated could be classified into a Gene Ontology category more often than expected by chance. A functional group with a high enrichment score was considered the leading group.

### Statistical analysis of genome-wide gene expression profiling

Differential gene expression analysis was carried out using the Partek Genomic Suite 6.6 software with three-way ANOVA analysis. The expression data were normalized using quantile normalization and robust multi-array analysis (RMA) background correction. The differentially expressed genes were reported to be significant in the cancer group when the fold-change was >2.0 and P-value with FDR was <0.05. The batch effect was removed as a source of variation.

### Sub-analysis of methylation and expression profile

The analysis was performed using the Partek Genomic Suite 6.6 software. We used the Pearson (linear) correlation to determine the correlation of our data. The correlated genes were considered significant at P<0.05.

### Integration of methylation and expression profile statistical analysis

Overlapped genes were identified based on significant gene symbols from the methylation and expression datasets. Datasets were imported into the MySQL relational database for downstream data analysis. The MySQL database allows rapid and accurate data filtering across different datasets. The datasets were compared in the pair-wise manner. Unique gene symbols identified between the overlapping comparisons were used in downstream analysis. Along with the unique gene list, methylation and expression values were extracted from the datasets for chromosome mapping, circular map generation and KEGG Pathway mapping. Mapping of the integrated gene list allowed visualization of overlapping genes on chromosome map overview, circular map overview and also KEGG pathway maps.

### Validation of genes using MS-MLPA

A total of four significant genes (*SFRP2*, *BTG4*, *APC* and *GPX7*) were selected from the DNA methylation profile for validation purpose. Validation was carried out using the MS-MLPA and followed the manufacturer’s instructions. The primers were designed and customized following the guidelines from MRC Holland (MRC-Holland, Amsterdam, The Netherlands). The amplification PCR product was carried out by using the 3500 Genetic Analyzer (Applied Biosystems, Foster City, CA, USA). The electrophoresis result was assessed by using the Coffalyser software version 1.0.0.43 (MRC-Holland). For the analyses, dataset of MS-MLPA was analyzed by the method described in a previous study (27). To quantify the methylation status of each of the genes, the probe relative peak area ratio of the digested sample was compared with that of the undigested samples. The digested sample with the probes of a relative peak area ratio of ≥0.25 was defined as methylated.

## Results

### Principal component analysis and sources of variation determine the quality of the microarray studies

**Figure 1:**
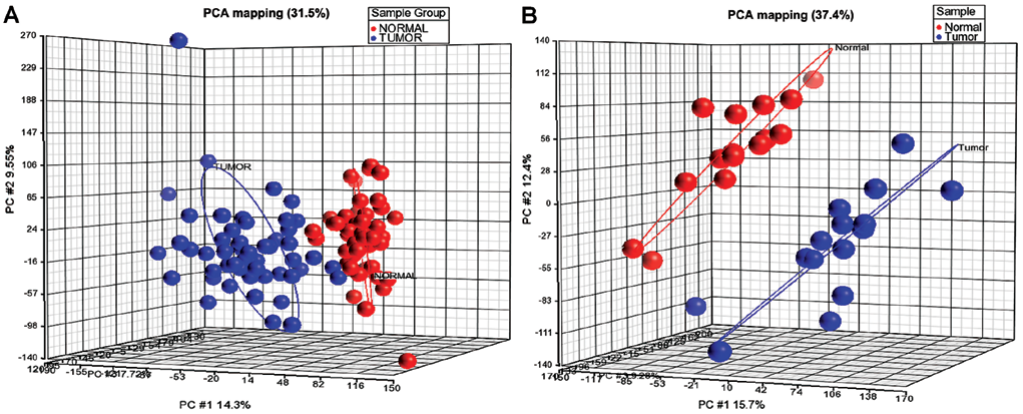
PCA mapping of (A) methylation and (B) expression profiling. Normal group (indicated by red color) was clustered distinctly from tumor group (indicated by blue color).

Principal component analysis (PCA) showed that the normal group (indicated by red color) was clustered distinctly from the tumor group (indicated by blue color) from the profiling data (Fig. 1). For the methylation study, one normal and one tumor sample were removed since the samples were classified in the opposite group. By applying three-way ANOVA, sources of variation were generated. The ’Sentrix barcode’ factor (for methylation study) and ‘Scan date’ (for expression study) factor were removed to avoid the batch effects.

### Genome-wide DNA methylation profiling of CRC in matched samples

The methylation profiling involved 110 CRC samples together with their neighboring non-cancerous colonic cells. The mean age for all patients was 60.4±12.83 years (Table I). The methylation chip analyses revealed a total of 27,578 CpG loci, covering 14,495 consensus coding sequences with an average of 1.9 CpG loci per sequence. We detected for each sample, on average, 27,506.64 loci at P<0.05 and 27,444.05 loci at P<0.01. A locus was considered to be detected at the two cut-off levels when the mean signal intensity from multiple probes for a particular CpG locus was significantly higher than the negative control on the same chip.

**Figure 2:**
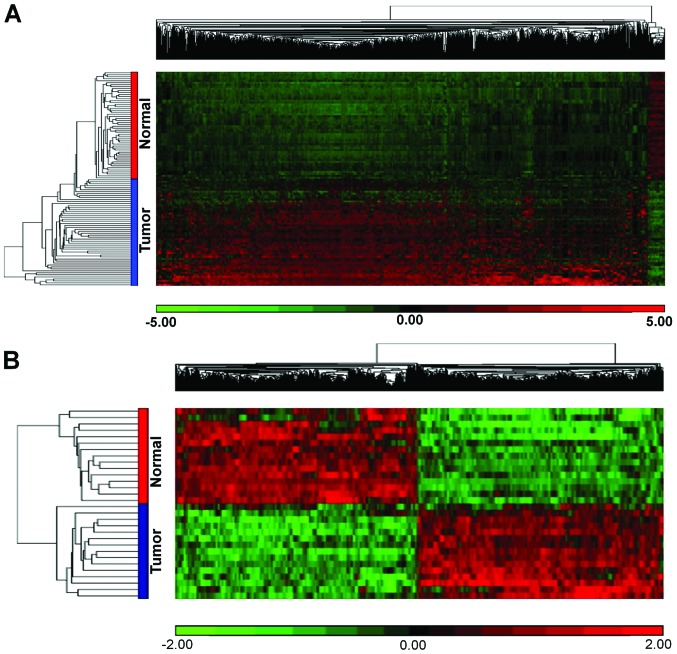
Supervised hierarchical clustering of methylation and expression profiling. (A) Supervised hierarchical clustering of methylation profile revealed 1,081 hypermethylated loci (red) and 36 hypomethylated loci (green). (B) Supervised hierarchical clustering of gene expression profile 709 genes were upregulated (red) and 699 genes were downregulated (green).

A total of 1,123 loci (845 genes) were found to be differentially methylated in CRC compared with the normal colonic epithelial samples. These loci were further classified into 1,081 hypermethylated loci (804 genes), 36 hypomethylated loci (36 genes) and 6 sex-chromosome methylated loci (5 genes). Sex-chromosomes loci were eliminated for the subsequent analysis to avoid bias to the present study. This is due to the methylation involved in the X-inactivation process that silences one out of the two copies of X chromosome in female in order to compensate for the gene dosage effect. Supervised hierarchical clustering of the significant differentially methylated loci in our study is shown in Fig. 2A. The top 10 highly significant hypermethylated loci were protein kinase, cAMP-dependent, regulatory, type I, β (*PRKAR1B*), cannabinoid receptor interacting protein 1 (*C2orf32*), zinc finger protein 542 (*ZNF542*), KH domain containing, RNA binding, signal transduction-associated 3 (*KHDRBS*), interferon regulatory factor 4 (*IRF4*), tissue factor pathway inhibitor 2 (*TFPI2*), potassium voltage-gated channel, KQT-like subfamily, member 5 (*KCNQ5*), filamin-binding LIM protein 1 (*FBLIM1*), eyes absent homolog 4 (*EYA4*) and spastic paraplegia 20 (*SPG20*). The top 10 most significantly hypomethylated loci were Fc receptor-like 3 (*FCRL3*), Granzyme K (Granzyme 3; Tryptase II) (*PRSS1*), bactericidal/permeability-increasing protein (*BPI*), v-akt murine thymoma viral oncogene homolog 3 (*AKT3*), pipecolic acid oxidase (PIPOX), peptidase inhibitor 3 (*PI3*), bactericidal/permeability-increasing protein-like 3 (*BPIL3*), solute carrier family 26, member 4 (*SLC26A4*), long intergenic non-protein coding RNA 152 (*MGC4677*) and defensin, β 119 (*DEFB119*).

### Genome wide expression profiling of CRC identifies differentially expressed genes in the same group of patients

The data of the genome wide expression profiling have been recently reported (28). Statistical analysis following normalization of data identified 1,408 differentially expressed genes in CRC compared to the normal samples. Supervised hierarchical clustering clearly showed a total of 709 genes were upregulated and 699 were downregulated genes (Fig. 2B). The top 10 most significant differentially upregulated genes were claudin 1 (*CLDN1*), phosphoribosyl pyrophosphate amidotransferase (*PPAT*), tumor protein D52-like 2 (*TPD52L2*), serine hydroxymethyltransferase 2 (*SHMT2*), cell division cycle associated 7 (*CDCA7*), chaperonin containing TCP1, subunit 3 (*CCT3*), eukaryotic translation initiation factor 2, subunit 2 β (*EIF2S2*), thyroid hormone receptor interactor 13 (TRIP13), inhibin, β A (*INHBA*) and negative elongation factor complex member C/D (*TH1L*). The top 10 most significant differentially downregulated genes were chromosome 2 open reading frame 88 (*C2orf88*), alcohol dehydrogenase 1B (*ADH1B*), erythrocyte membrane protein band 4.1-like 4A (*EPB41L4A*), transmembrane and immunoglobulin domain containing 1 (*TMIGD1*), serum/glucocorticoid regulated kinase 1 (*SGK1*), histone deacetylase 9 (*HDAC9*), UDP-glucose pyrophosphorylase 2 (*UGP2*), sodium channel, voltage-gated, type IX, α subunit (*SCN9A*), membrane-spanning 4-domains, subfamily A, member 12 (*MS4A12*) and solute carrier family 4, sodium bicarbonate cotransporter, member 4 (*SLC4A4*).

### Methylation specific-multiple ligation probe amplification (MS-MLPA) confirmed the methylation profiling results

**Figure 3:**
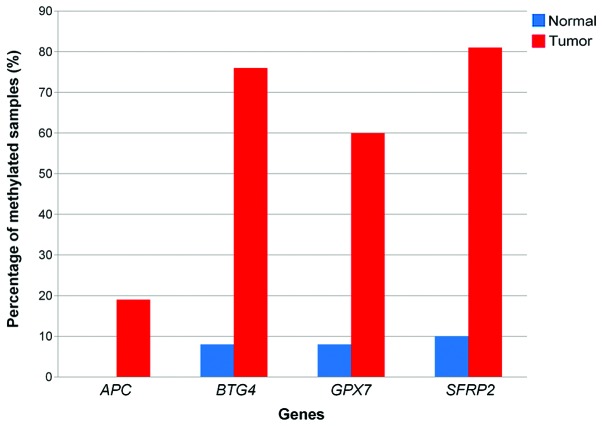
MS-MLPA analysis. MLPA-analysis revealed the percentage of methylation in tumor samples was higher than normal samples. The red bar on the figure shows tumor samples whereas the blue bar shows normal samples. The *SFRP2* gene has the highest percentage of methylation in tumor samples with 81% followed by *BTG4* (76%), *GPX7* (60%) and *APC* (19%).

Four hypermethylated genes, i.e., secreted frizzled-related protein 2 (*SFRP2*), BTG4, adenomatous polyposis coli (*APC*) and glutathione peroxidase 7 (*GPX7*), to validate the methylation profiling data. Data analysis of MS-MLPA showed high methylation for all four validated genes in CRC compared to the normal samples (Fig. 3).

### Sub-analysis of methylation and gene expression profiling of 15 pair-matched samples

**Figure 4:**
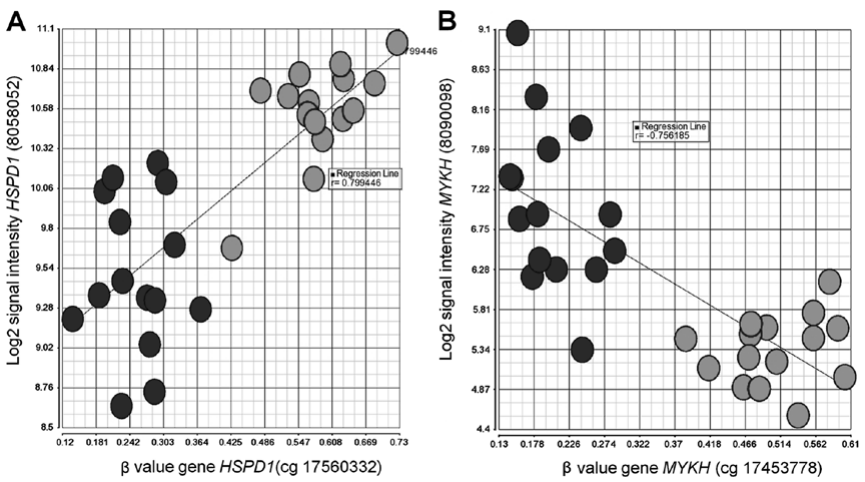
Correlation analysis of methylation and expression profiles. Positive and negative correlation of 15 pairs matched samples. Dark dots are normal samples whereas light dots are tumor samples. (A) Positively correlated gene *HSPD1* (r=0.7994) and (B) negatively correlated gene *MYKH* (r=−0.7562) were shown. Tumor and normal samples are differentially methylated or expressed in both genes.

We correlated the data at P<0.05 and identified 188 significant loci (covered by 136 genes) from the two profiles including 83 negatively (66 genes) and 105 positively correlated loci (70 genes). The top 5 positively correlated genes were heat shock 60 kDa protein 1 (*HSPD1*) (r= 0.799443) (Fig. 4A), *SLC4A4* (0.77751), family with sequence similarity 5, member C (*FAM5C*) (0.777301), partner of NOB1 homolog (*PNO1*) (0.769136) and CD163 molecule-like 1 (*CD163L1*) (0.766757). The top 5 negatively correlated genes were myosin light chain kinase (*MYLK*) (−0.756185) (Fig. 4B), nuclear receptor subfamily 5, group A, member 2 (*NR5A2*) (−0.738724), transforming growth factor, β-induced (*TGFBI*) (−0.674433), von Willebrand factor A domain containing 5A (*VWA5A*) (−0.673862) and D-tyrosyl-tRNA deacylase 1 (*DTD1*) (−0.652264).

### Integrated analysis reveals 32 important genes in CRC

**Figure 5:**
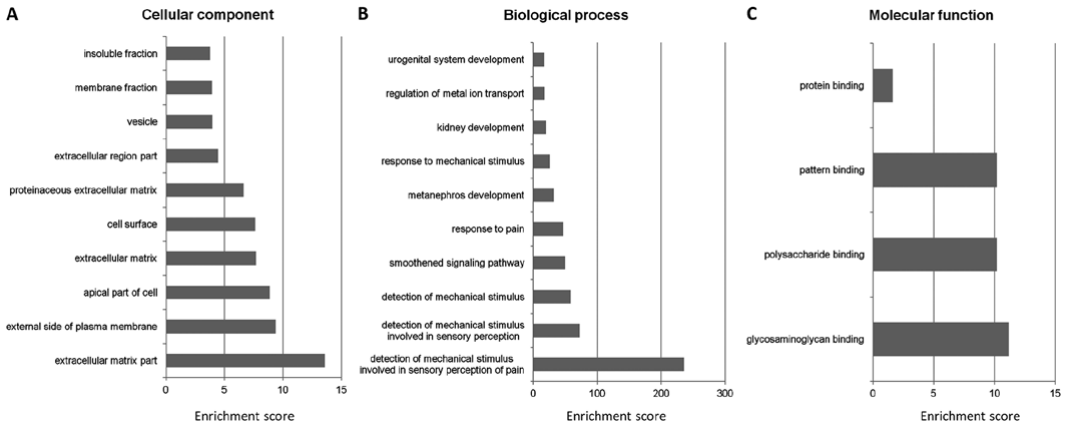
GO enrichment analysis of integration profile. GO enrichment analysis of 32 overlapping genes revealed the enriched (A) cellular component, (B) biological process and (C) molecular function. The number is the enrichment score. The high enrichment score shows the genes found more frequently in a particular group.

**Figure 6:**
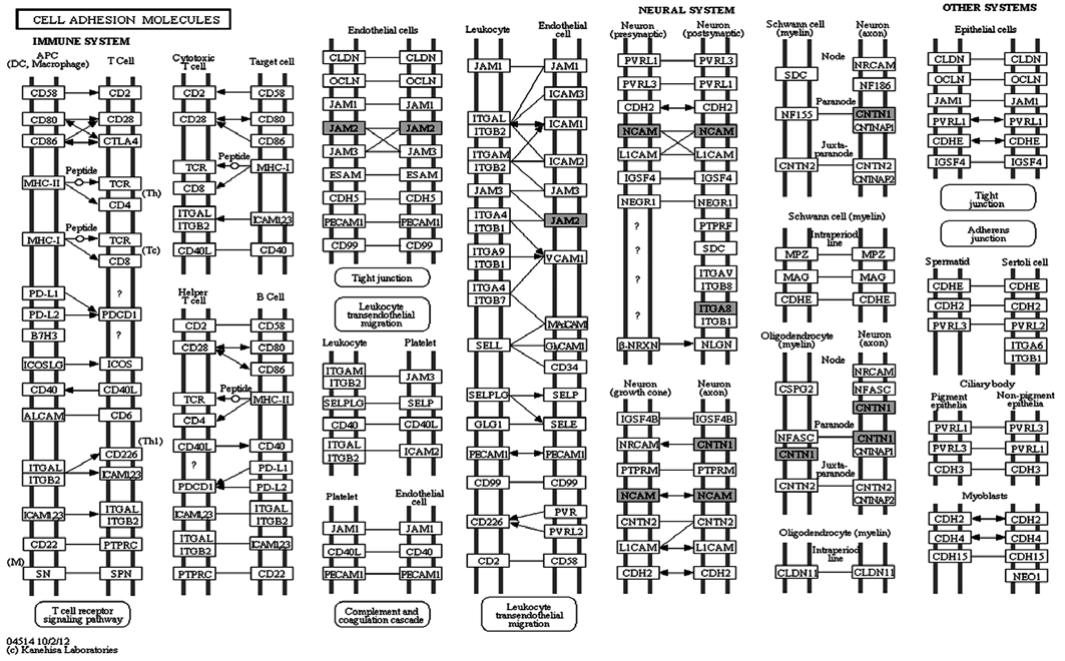
Cell adhesion molecules (CAMs) pathway. CAMs pathway (hsa04514) is an enriched pathway in the integration profile with P-value 0.0122. The highlighted genes were the genes found in our integration profile.

We identified 32 significant overlapping genes from the methylation and gene expression profiles (Table II). Most of the significant genes (27 genes) have a negative association (hypermethylation and downregulation) and only 5 genes showed a positive association (hypermethylation and upregulation or hypomethylation and downregulation). We then used the Gene Ontology (GO) enrichment analysis to classify the genes into the categories of cellular component, biological process and molecular function. Under the cellular component category, the integrated genes were highly enriched in the extracellular matrix part [Enrichment score (ES), 13.60] (Fig. 5A). For the biological process and molecular function categories, significant genes were highly enriched in the wide group of detection of mechanical stimulus (ES=235.27) (Fig. 5B) and glycosaminoglycan binding (ES=11.19) (Fig. 5C), respectively. Using the Kyoto Encyclopedia of Genes and Genomes (KEGG) database, we identified a total of 95 pathways and 11 of these pathways were associated with the CRC. These includes well-documented pathways in CRC including the cell adhesion molecule, Hedgehog signaling, phosphatidylinositol 3′-kinase (PI3K)-Akt signaling, focal adhesion, pathways in cancer, basal cell carcinoma, colorectal cancer, Wnt signaling, Janus kinase/signal transducers and activators of transcription (JAK-STAT) signaling, transcriptional misregulation in cancer and mitogen-activated protein kinase (MAPK) signaling pathway. Only one pathway was found to be enriched (P<0.05) in our data, the cell adhesion molecules (CAMs) (Fig. 6). Significant genes detected in this pathway were *JAM2*, *NCAM1*, *ITGA8* and *CNTN1*.

### Circular map shows the chromosome distribution of three profiles

**Figure 7:**
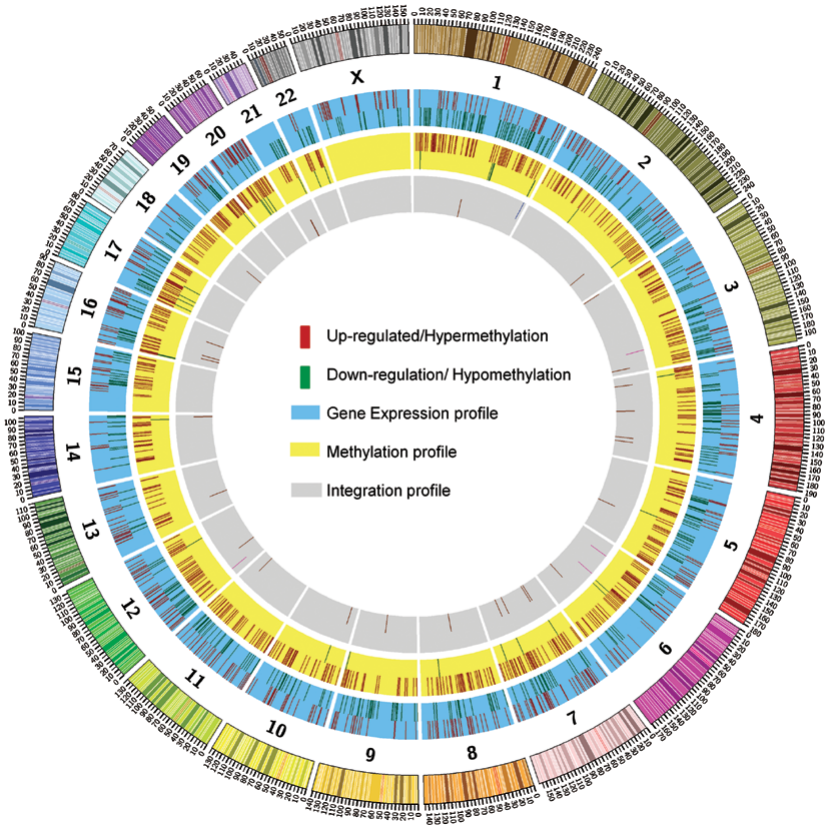
Circular map of chromosome distribution. Circular map shows the distribution of genes in each chromosome of methylation, gene expression and integration profiles. The outermost circular ring is the chromosome.

We analyzed the frequency of the methylated loci, the upregulated or downregulated genes and the integrated genes in each chromosome. Fig. 7 shows the distribution of genes for each profile. We found that the genes with hypermethylated promoters in CpG islands and also those that were differentially expressed were generally identified in all chromosomes. Chromosome 1 has the highest frequency of the hypermethylated loci (91 loci) followed by chromosome 7 (78 loci), chromosome 19 (77 loci) and chromosome 6 (73 loci). Conversely, chromosome 20 has the highest frequency of the hypomethylated loci (7 loci) followed by chromosome 1 (6 loci), chromosome 17 (4 loci) and chromosome 7 (3 loci). The integrated genes were distributed in all the chromosomes.

We identified four clusters of genes when we examined closely the distribution of methylated genes in each chromosome. These clusters were located in chromosome 6, 16 and 19 and belonged to specific gene families. One cluster was within the HIST1H family [histone cluster 1, H2bb (*HIST1H2BB*) with histone cluster 1, H3c (*HIST1H3C*) and histone cluster 1, H3f (*HIST1H3F*) with histone cluster 1, H3g (*HIST1H3G*), another in the FOX family (forkhead box F1 (*FOXF1*), fork-head box F1 (*FOXC2*) and forkhead box FL (*FOXL1*)] and two large clusters in the zinc finger (ZNF) family. For the gene expression study, 16 clusters were distributed across the chromosomes including one single cluster which encompassed >10 genes. This cluster included the metallothionein 2A (*MT2A*), nucleoporin 93 kDa (*NUP93*) and 8 other genes derived from the metallothionein 1 (*MT1*) family.

## Discussion

The low sensitivity and specificity of the current serum-based markers in the detection and prognostication of CRC have rendered the identification of new candidates crucial. The emergence of methylation biomarkers for CRC has important implications since the methylation events are reversible. However, methylation markers derived from a single profiling are not sufficient to reveal the overall mechanism involved in CRC. To overcome this issue, the integrative approach combining different genomic profiling analyses could provide new insights into the biology of the CRC and help in identifying potential diagnostic markers.

To show the validity of our results, we correlated our findings with those of a recent methylation study conducted in 2010 which involved 91 cancer and 28 normal samples (21). Our results showed a higher number of hypermethylated genes with 804 genes compared to the 132 genes reported in that study. Overlapping our data with theirs revealed 59 genes, 7 of which were in our top 50 significant gene list based on P-values (21). We then compared our data with those from a Korean study that used 22 paired colorectal cancer and adjacent normal mucosa (29). We found 16 hypermethylated genes overlapped between our data and their top 20 hypermethylation genes with 10 genes appearing on our top 50 significant gene list. These included alcohol dehydrogenase, iron containing, 1 (*ADHFE1*), bol, boule-like (*BOLL*), cut-like homeobox 2 (*CUTL2*), *KCNQ5*, protocadherin γ subfamily C, 4 (*PCDHGC4*), *PRKAR1B*, solute carrier family 6 (neutral amino acid transporter), member 15 (*SLC6A15*), *SPG20*, *TFPI2* and unc-5 homolog C (*UNC5C*) (29). The *ADHFE1*, *BOLL*, *SLC6A15* and *TFPI2* genes were validated using pyrosequencing (29).

Our integrative analysis correlating methylation and gene expression data revealed 27 genes with a negative association (hyper-down) and 5 with a positive association (hyper-up and hypo-down). Of the 27 hypermethylated genes with low gene expression, 18 were related to CRC including *GSTM2*, *ZNF655*, *MYH11*, *HHIP*, *RSPO3*, *RNF152*, *CAV1*, *SFRP2*, *JAM2*, *SPG20*, *TMEFF2*, *SST*, *SLIT2*, *SCNN1B*, *ADAMST1*, *GLI3*, *CKB* and *BMP3* (19,30–38). Of these, *CAV1*, *SFRP2*, *TMEFF2*, *SST* and *SLIT2* have been identified as tumor-suppressor genes (39–43). The remaining genes were associated with other cancer types. *CHL1* and *ITGA8* were identified as specific potential biomarkers for renal and ovarian cancer, respectively (44,45).

*SFRP2* is a dominant-negative inhibitor in the Wnt signaling pathway that is involved in regulating cell proliferation, migration, and differentiation (46,47). Hypermethylation of *SFRP2* that resulted in the downregulation of *SFRP2* was detected via methylation-specific PCR (MSP) and quantitative PCR (qPCR) in colorectal carcinoma as compared to adenoma (48). Another study reported that hypermethylation of *SFRP2* in stool DNA may be a potential biomarker in the detection of CRC (31). Another gene, *SPG20*, encodes Spartin, which is a multifunctional protein that plays a vital role in the turnover of lipid droplet and intracellular epidermal growth factor receptor trafficking (49,50). Hypermethylation of *SPG20* downregulates Spartin and may lead to cytokinesis arrest, which in turn is associated with carcinogenesis (33). A recent study showed that SPG20 has 80.2% sensitivity and 100% specificity in detecting colorectal cancer in stool samples using the MSP approach (22).

We also identified the *COLI2A1*, *MME*, *HIST1H3B* and *GRIN2B* genes, which were hypermethylated with a high expression. Hypermethylation in the promoters of *MME* and *GRIN2B* were previously reported to contribute to Alzheimer’s disease and seizures, respectively (51,52). In the integrated list of genes, *AKT3* was the only gene with hypomethylation and a low gene expression. These data were supported by a study on hepatocellular carcinoma in which the gene was also found to be hypomethylated (53). In a separate study, the specific knock-down of *AKT3* reduced the level of phosphorylated Akt and inhibited cell growth in malignant melanoma (54). The low mRNA level of *AKT3* was associated with a higher grade of malignant glioma (55). The positive association exhibited for *AKT3* has suggested that there is possibly another layer of regulation. One of the possible explanations for this phenomenon is the existence and influence of long non-coding RNAs (lncRNA). Functional lncRNA may act as an activator or repressor for the expression of genes at the post-transcriptional level via mechanisms such as alternative splicing, influencing RNA polymerase binding efficiency and even by modification of epigenetic state of the gene (56–59).

From the integrative analysis, we found that 11 out of 95 KEGG pathways were associated with colorectal carcinogenesis. The most enriched pathway identified in our data was cell adhesion molecules, covered by the integrated genes *JAM2*, *NCAM1*, *ITGA8* and *CNTN1*. CAM plays a vital physical function in defining the multicellular organism’s structure and signaling as well (60,61). It is known to facilitate intercellular adherence and is able to promote cell invasion, motility and migration (62). CAMs such as L1-CAM and neurone glial-related (Nr)-CAM have been shown to be associated with the Wnt signaling pathway and their overexpression has been associated with poor prognosis (63,64). To the best of our knowledge, there is no data available describing the methylation status of the CAMs genes with the exception of *JAM2*. One study reported an inverse association between methylation and the gene expression of *JAM2* in pre-malignant and malignant colorectal tissues (32). This observation supported our findings on *JAM2*, which is involved in cell-cell adhesion and cell-environment interaction.

In conclusion, our integrative analysis which combined DNA methylation and gene expression profiling datasets revealed a potential panel of biomarkers for the diagnosis or prognosis of colorectal cancer. Our integrative analysis also revealed a list of novel genes not previously reported in colorectal cancer.

## Figures and Tables

**Table I:** Distribution of clinicopathological characteristics of 55 paired matched samples.

Characteristics	No.	(%)
Gender		
Male	22	40.00
Female	33	60.00
Age (years)		
<50	9	16.36
>50	46	83.64
Ethnicity		
Malay	27	49.09
Chinese	25	45.46
India	3	5.45
Duke’s staging		
A	4	7.27
B	31	56.36
C	20	36.36
Differentiation		
Well differentiated	31	56.36
Moderately differentiated	21	38.18
Poorly differentiated	3	5.46
Location		
Right	13	23.64
Left	42	76.36

**Table II:** Integrated gene list.

No.	Gene symbol	Gene name	Methylation profile					Gene expression profile			
			Probeset ID	Methylation level	Fold-change	P-value	β-value	TranscriptID	Expression level	Fold-change	P-value
1	*SPG20*	Spastic paraplegia 20	cg18755783	Hyper	4.759	3.07E-37	0.4063	7970999	DR	−2.091	1.19E-04
2	*JAM2*	Junctional adhesion molecule 2	cg03382304	Hyper	5.529	1.60E-27	0.4061	8068024	DR	−3.286	5.40E-07
3	*GSTM2*	Glutathione S-transferase *μ* 2	cg16670497	Hyper	4.874	1.16E-29	0.3630	7903753	DR	−2.261	3.00E-05
4	*SFRP2*	Secreted frizzled-related protein 2	cg23207990	Hyper	2.560	1.37E-25	0.3079	8103254	DR	−2.781	2.61E-03
5	*CNTN1*	Contactin 1	cg27352992	Hyper	2.590	3.79E-23	0.2639	7954899	DR	−2.159	5.56E-06
6	*CHL1*	Close homolog of L1	cg00903242	Hyper	2.331	4.06E-22	0.2576	8077270	DR	−2.394	2.08E-06
7	*TMEFF2*	Transmembrane protein with EGF-like and two follistatin-like domains 2	cg18221862	Hyper	3.020	1.77E-21	0.2975	8057803	DR	−2.520	3.47E-04
8	*ITGA8*	Integrin, α 8	cg16902509	Hyper	2.083	8.70E-19	0.2631	7932254	DR	−2.312	4.31E-04
9	*SST*	Somatostatin	cgl3206017	Hyper	2.093	1.78E-17	0.2270	8092682	DR	−2.183	3.14E-06
10	*SLIT2*	Slit homolog 2	cg18972811	Hyper	2.462	4.94E-17	0.2667	8094301	DR	−2.075	2.92E-05
11	*MAMDC2*	MAM domain containing 2	cg11656547	Hyper	2.457	2.03E-15	0.2636	8155754	DR	−3.061	1.32E-05
12	*SCNN1B*	Sodium channel, non-voltage-gated 1, β subunit	cg23113963	Hyper	2.129	1.14E-14	0.1993	7994074	DR	−4.948	8.53E-06
13	*ZNF655*	Zinc finger protein 655	cgl3636404	Hyper	6.100	5.59E-14	0.2163	8134631	DR	−2.882	3.72E-06
14	*ADAMTS1*	ADAM metallopeptidase with thrombospondin type 1 motif, 1	cg00472814	Hyper	3.048	1.53E-11	0.2377	8069676	DR	−2.609	3.22E-05
15	*CKB*	Creatine kinase, brain	cg05786809	Hyper	2.642	1.73E-10	0.1458	7981427	DR	−3.186	1.34E-04
16	*HHIP*	Hedgehog interacting protein	cg13749822	Hyper	2.012	2.48E-10	0.1798	8097628	DR	−2.487	1.99E-05
17	*ELMOl*	Engulfment and cell motility 1	cg08453021	Hyper	6.708	3.66E-10	0.2530	8139057	DR	−2.117	6.49E-04
18	*RSPO3*	R-spondin 3	cg09979256	Hyper	13.015	1.31E-09	0.2102	8121916	DR	−2.148	4.85E-03
19	*TRPA1*	Transient receptor potential cation channel, subfamily A, member 1	cg0l610488	Hyper	2.630	1.46E-08	0.2160	8151341	DR	−2.089	1.56E-03
20	*GNAO1*	Guanine nucleotide binding protein (G protein), α activating activity polypeptide O	cg21530453	Hyper	2.839	1.49E-08	0.1349	7995739	DR	−2.314	3.25E-04
21	*MYH11*	Myosin, heavy chain 11, smooth muscle	cg17880199	Hyper	3.620	1.48E-07	0.1470	7999674	DR	−7.642	2.08E-05
22	*CAMK4*	Calcium/calmodulin-dependent protein kinase IV	cg05497616	Hyper	7.229	3.85E-06	0.1225	8107307	DR	−2.277	2.95E-06
23	*RNF152*	Ring finger protein 152	cg07980518	Hyper	2.322	3.94E-06	0.0872	8023598	DR	−5.566	2.46E-08
24	*NCAM1*	Neural cell adhesion molecule 1	cg20268522	Hyper	2.569	3.74E-05	0.1095	7943892	DR	−2.212	2.10E-05
25	*CAV1*	Caveolin 1, caveolae protein, 22 kDa	cg22126032	Hyper	2.252	0.0003646	0.0597	8135594	DR	−2.289	6.45E-03
26	*BMP3*	Bone morphogenetic protein 3	cgO1049530	Hyper	2.010	0.0030589	0.0817	8096070	DR	−5.583	2.15E-07
27	*GLI3*	GLI family zinc finger 3	cg09405612	Hyper	2.591	0.0046925	0.0414	8139212	DR	−2.099	1.94E-04
28	*COL12A1*	Collagen, type XII, α 1	cg08009622	Hyper	3.118	2.27E-14	0.2118	8127563	UR	3.367	2.95E-05
29	*MME*	Membrane metallo-endopeptidase	cg16580737	Hyper	8.756	5.00E-08	0.1904	8083494	UR	3.512	2.13E-03
30	*HIST1H3I*	Histone cluster 1, H3i	cgl2181621	Hyper	2.737	0.0002427	0.1142	8124531	UR	2.329	2.95E-03
31	*GRIN2B*	Glutamate receptor, ionotropic, N-methyl D-aspartate 2B	cgl3264741	Hyper	2.208	0.0017283	0.0518	7961422	UR	2.145	1.10E-04
32	*AKT3*	v-akt murine thymoma viral oncogene homolog 3 (protein kinase B, *γ*)	cgl1314684	Hypo	−2.298	6.96E-32	−0.1808	7925531	DR	−2.261	2.49E-03

Hyper, hypermethylation; Hypo, hypomethylation; DR, downregulation; UR, upregulation.

## Abbreviations

ANOVA: analysis of variance
CAMs: cell adhesion molecules
CEA: carcinoembryonic antigen
CG1: CpG islands
CIMP: CpG island methylator phenotype
ES: enrichment score
FDR: false discovery rate
GO: Gene Ontology
KEGG: Kyoto encyclopedia of genes and genomes
lncRNA: long non-coding RNAs
MBDs: methyl-CpG binding proteins
MS-MLPA: methylation specific-multiple ligation probe amplification
MSP: methylation-specific PCR
PCA: principal component analysis
PCR: polymerase chain reaction
qPCR: quantitative PCR
RIN: RNA integrity number
